# An Emerging Treatment of Viral Warts: A Case Report of a Patient Treated With Intralesional Immunotherapy

**DOI:** 10.7759/cureus.92122

**Published:** 2025-09-12

**Authors:** Azzam A Al Marbouai, Raqiya Al Rajaibi

**Affiliations:** 1 Dermatology, Sultan Qaboos University Hospital, Muscat, OMN; 2 Dermatology, Rustaq Extended Health Center, Rustaq, OMN

**Keywords:** common warts, dermatology, human papilloma virus, intralesional immunotherapy, mmr vaccine, oman, viral warts

## Abstract

Viral warts are common epidermal growths caused by human papillomavirus (HPV), causing a wide range of diseases ranging from benign lesions to invasive tumours. The current treatment options for warts include topical agents, destructive methods, and surgical excision. Here, we present a 45-year-old male patient with no significant past medical history, who presented with a few weeks' history of numerous skin-coloured to hyperpigmented papules along the beard bilaterally. A clinical diagnosis of facial warts was made, as some had verrucous surfaces. The patient was treated with three sessions of intralesional (IL) immunotherapy with measles-mumps-rubella (MMR) vaccine, with two-week intervals between the IL sessions. There was a significant improvement after the first session, and it was completely cleared after the third session with no side effects. An extended follow-up for around two years showed no recurrence of warts. Multiple IL immunotherapies have been studied. IL immunotherapy with the MMR vaccine seems safe and effective, with more cosmetically acceptable results for facial wart treatment. There are no clear guidelines on when to use immunotherapy, but it is generally tried for extensive, recurrent, and refractory warts and difficult-to-treat areas.

## Introduction

Viral warts are common epidermal growths caused by human papillomavirus (HPV) [1]. Papillomaviruses can infect human skin and mucosal surfaces [2], causing a wide range of diseases ranging from benign lesions to invasive tumours [3].

The current treatment options include topical agents (e.g., salicylic acid, imiquimod cream, podophyllin, and podophyllotoxin); destructive methods (e.g., cryotherapy, electrocautery, CO2 laser, trichloroacetic acid (TCA), and surgical excision [4-6]. Many of these options are destructive and may result in scarring, while less aggressive approaches can lead to lesion recurrence [7].

With new advances in treatment modalities, intralesional (IL) immunotherapy has emerged as a promising treatment option in cases of multiple lesions. The specific IL immunotherapies studied include *Candida albicans*, measles-mumps-rubella (MMR), *Trichophyton*, and tuberculin antigens such as purified protein derivative (PPD) and Bacillus Calmette-Guérin (BCG) [8].

In the patient presented in this case report, IL immunotherapy was selected due to the presence of multiple, recurrent lesions that were resistant to conventional destructive treatments, and the patient’s preference to avoid further procedures that could result in scarring. This report demonstrates the successful use of IL immunotherapy in treating viral warts.

## Case presentation

**Figure 1:**
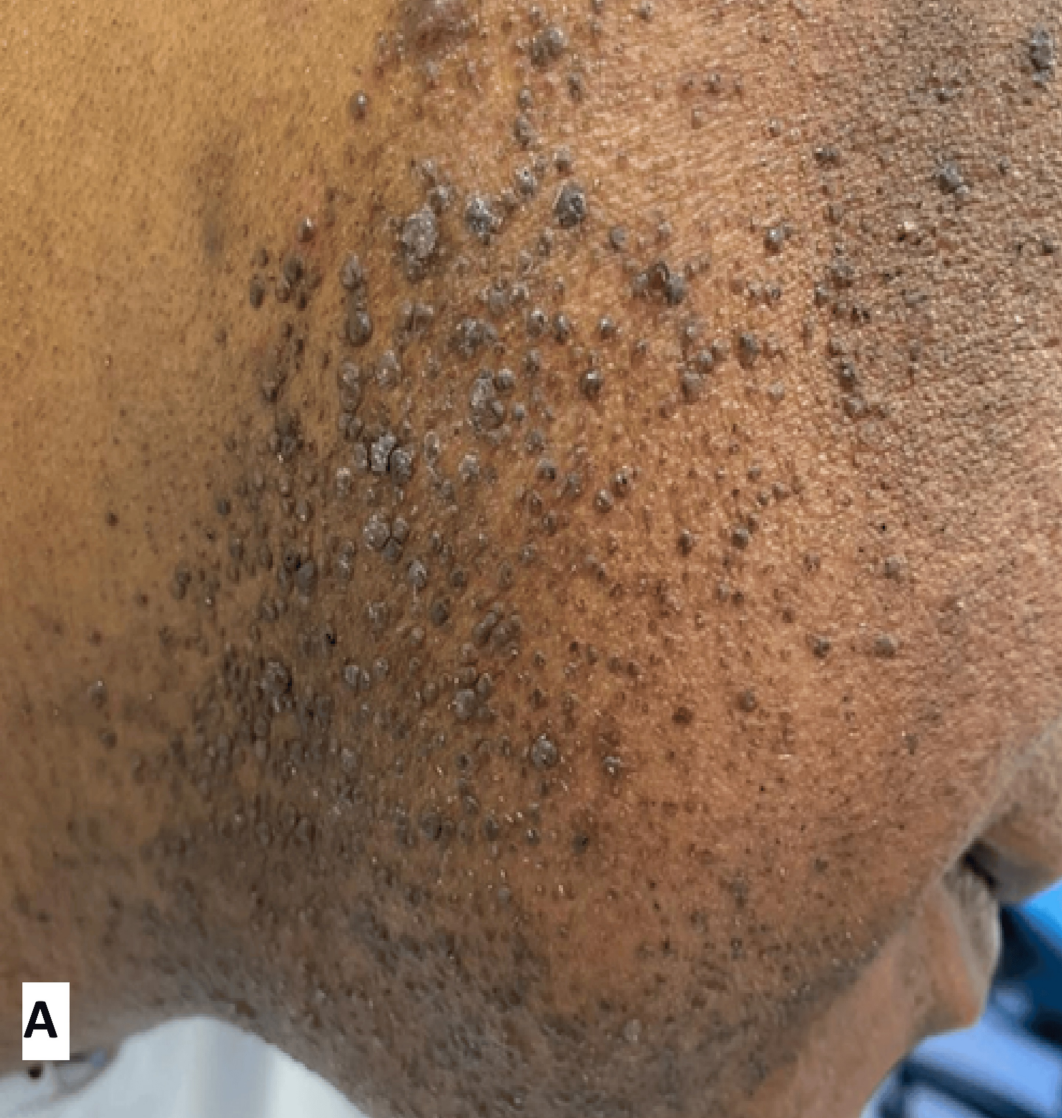
Numerous papules (ranging from skin coloured to hyperpigmented) along the beard area of the face; some have a verrucous surface

A 45-year-old male patient, with no significant medical and surgical background and not on any medication, presented with multiple papules over the face for a few weeks. On examination, the patient was found to have numerous skin-coloured to hyperpigmented papules along the beard area of the face, some of which had verrucous surfaces (Figure 1). The diagnosis of facial warts was made by an experienced dermatologist based on the typical morphology and distribution of lesions. Histopathology was not performed, as the clinical features were characteristic, and invasive testing was not deemed necessary.

The patient was initially started on salicylic acid for three months with no improvement, and then shifted to cryotherapy for three sessions in five months, which did not show acceptable results, alongside the side effects of cryotherapy. After that, we discussed IL immunotherapy (MMR) with the patient. Written informed consent was obtained prior to the initiation of IL immunotherapy, including an explanation of the procedure, benefits, and possible side effects.

**Figure 2:**
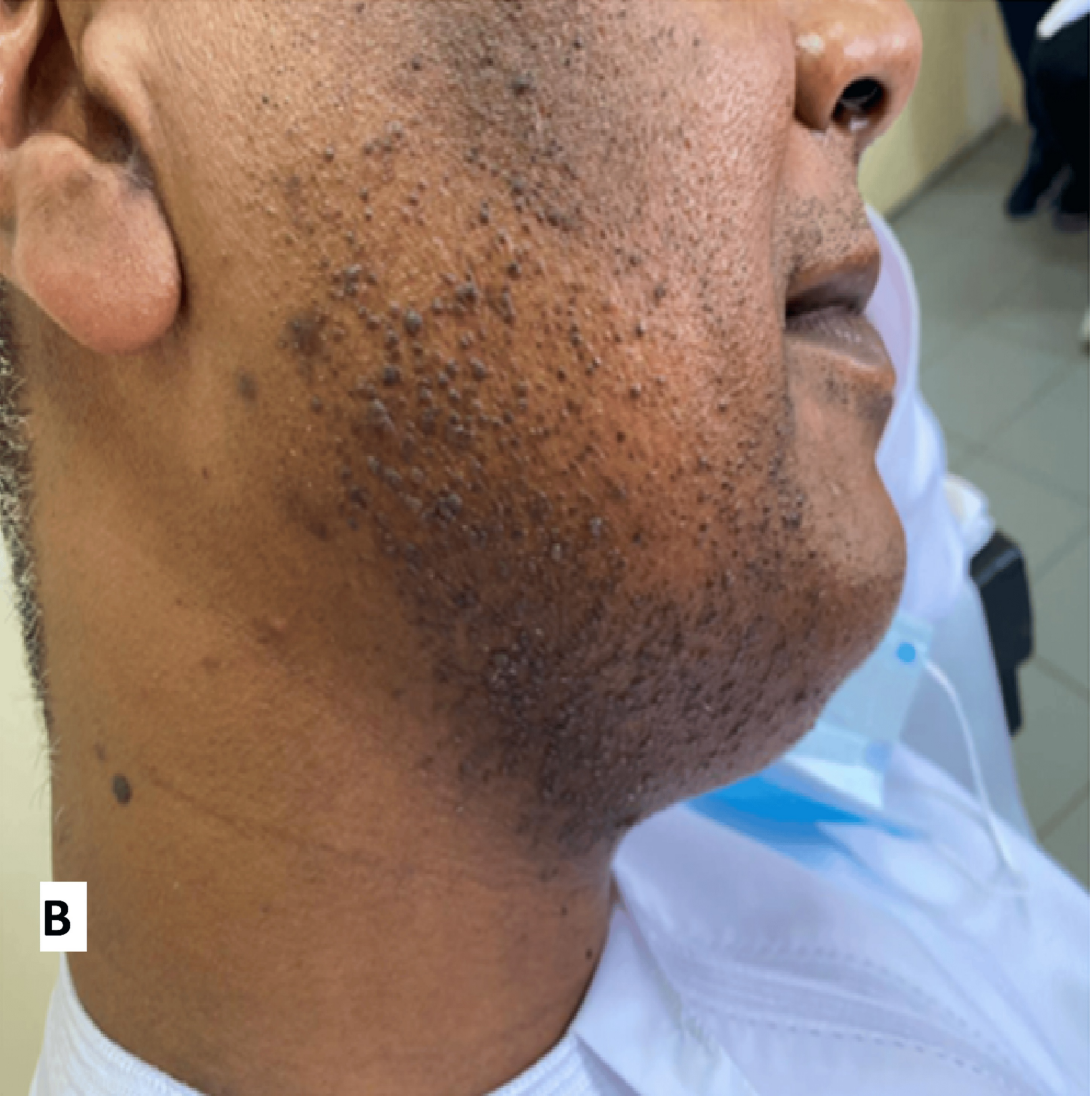
Two weeks post first session with single intralesional injection of MMR vaccine. MMR: measles-mumps-rubella

**Figure 3:**
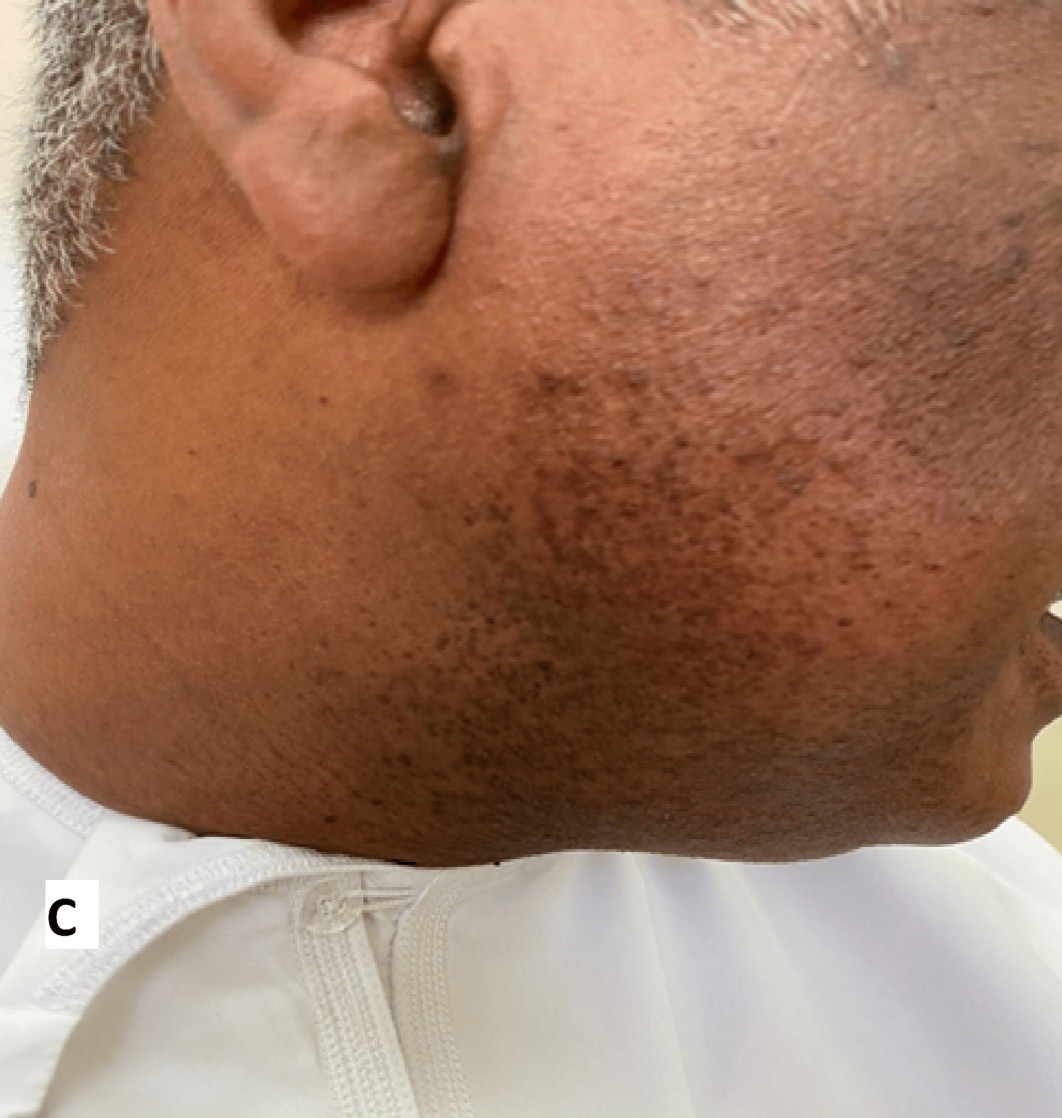
Photograph after the third session of intralesional immunotherapy, showing complete clearance.

The MMR vaccine was prepared with a dilution of 0.5 ml. The most considerable warts were injected with 0.1 ml each (a total of five warts on both sides). The patient received three sessions with two-week intervals between them. There was a significant improvement after the first session (Figure 2), and it almost cleared after the third session (Figure 3) with no side effects. The patient was evaluated for possible immunosuppression before initiating IL immunotherapy. A full clinical review and basic laboratory work-up (CBC, renal, liver function, and HIV screening) were performed, which were all within normal limits, ruling out any underlying immunodeficiency. An extended follow-up for around two years showed no recurrence of warts.

## Discussion

The clearance of HPV and subsequent resolution of warts depends on a cell-mediated immune response. IL immunotherapy is believed to enhance this response by delivering antigens directly to the wart site, thereby triggering a T cell-mediated systemic reaction. This form of immunotherapy has shown efficacy in pediatric and adult patients, especially in cases involving multiple or recalcitrant warts [9]. A two-case report published in 2020 from Oman showed a complete response after receiving a single IL injection of the MMR vaccine without requiring additional treatments [10]. Our patient experienced minimal pain and erythema for a few hours as a side effect, which has been reported in previous studies [10].

Previous studies using the MMR vaccine in an IL manner have yielded similar results, demonstrating optimal outcomes. A study evaluating the role of intradermal and IL (MMR) vaccines in the treatment of common warts showed that the majority of patients (57.6%) did not experience any adverse effects during the treatment and follow-up period; adverse effects, when present, were minimal and mainly consisted of pain at the injection site (45.5%) and itching (9.1%), which were more common in the group receiving IL treatment compared to intradermal treatment. In the intradermal MMR vaccine group, 60.6% did not respond to the treatment, whereas in the IL group, only 15.2% of patients did not show any clinical response [11].

The efficacy and safety of IL immune therapy, whether using the PPD or MMR vaccine, are similar when treating warts [12]. Immunotherapy is a cost-effective, highly effective, well-tolerated treatment option [8]. It has successfully cleared both treated and distant site lesions [9]. Compared to traditional treatment methods, IL immunotherapy with PPD or MMR is associated with fewer side effects, a lower recurrence rate [8], and there is an absence of scarring or pigmentation commonly seen with destructive wart therapies. An additional advantage of IL immunotherapy is its ability to stimulate a systemic immune response, thereby clearing untreated distant lesions, an outcome rarely achieved with local destructive therapies [13]. 

As a result, it can be recommended as the initial treatment choice for multiple and stubborn warts. However, it is important to clarify that while IL immunotherapy can be considered early in the treatment pathway for stubborn warts, it is usually introduced after conventional methods (such as topical agents and cryotherapy) have failed [14], as demonstrated in our patient. Patient selection for IL immunotherapy requires basic clinical and laboratory screening to exclude immunosuppression, as immunocompromised individuals may not mount an adequate immune response [15]. A full medical history and, when indicated, HIV screening and CBC should be done prior to treatment initiation.

However, conducting well-designed, placebo-controlled studies on a large scale is crucial to determine the minimum effective dose, dosing schedule, and duration of therapy required for an effective treatment regimen. These studies are necessary to provide reliable recommendations. Nevertheless, it is essential to note that patients may experience dissatisfaction and poor compliance with treatment if they do not observe the desired therapeutic response in the short term. Factors such as the long duration of treatment, slow response to immunotherapeutic agents, injection site pain, and the availability of internet-based information about alternative and potentially more effective treatment methods may contribute to a high dropout rate [5]. In our patient, no adverse effects such as injection site pain were reported. The injections were well tolerated and administered without the need for topical anaesthesia.

## Conclusions

Our findings indicate that the MMR vaccine is a safe and efficacious treatment for viral warts with no significant adverse effects. Notably, it offers an advantage over destructive therapies by avoiding scarring or disfigurement. Moreover, the IL administration demonstrated efficacy, suggesting it could be the preferred approach for treating representative warts. These conclusions are supported by the documented clinical improvement observed after three IL MMR sessions, with near-complete lesion clearance, absence of recurrence over two years, and no reported side effects in our patient.
